# Novel multiplex assay for profiling influenza antibodies in breast milk and serum of mother-infant pairs

**DOI:** 10.12688/f1000research.16717.2

**Published:** 2019-03-11

**Authors:** Kirsi M. Järvinen, Jiong Wang, Antti E. Seppo, Martin Zand

**Affiliations:** 1 Department of Pediatrics, University of Rochester Medical Center, Rochester, NY, 14642, USA; 2 Department of Medicine, Division of Nephrology, University of Rochester Medical Center, Rochester, NY, 14642, USA; 3 Rochester Center for Health Informatics, University of Rochester Medical Center, Rochester, NY, 14642, USA

**Keywords:** breast milk, influenza, infants, protection, transplacental, antibody

## Abstract

**Background:** During early life, systemic protection to influenza is passively provided by transplacental transfer of IgG antibodies and oral and gastrointestinal mucosal protection via breast milk (BM) containing predominantly IgA. Immune imprinting, influenced by initial exposure of the infant immune system to influenza, has recently been recognized as an important determinant of future influenza immune responses.

**Methods:** We utilized stored frozen BM from a prospective birth cohort to assess immune factors in human milk. The earliest available BM and a paired, timed serum sample was assessed from each of  7 mothers. Paired infant serum samples were assayed at up to three time points during the first 12 months of life, one prior to assumed disappearance of transplacentally transferred IgG, and one after. We utilized a novel multiplex assay to assess mothers’ and infants’ IgG and IgA antibodies in serum to a panel of  30 individual recombinant hemagglutinin (rHA) proteins of influenza virus strains and chimeric rHAs. We also characterized IgA and IgG antibody levels in breast milk which provide mucosal protection.

**Results:** Our pilot results, analyzing a small number of samples demonstrate the feasibility of this method for studying paired maternal-infant IgG and IgA anti-influenza immunity patterns. Unlike IgG antibodies, breast milk influenza virus HA-specific IgA antibody levels and patterns were mostly discordant compared to serum.  As expected, there was a steady decay of infant influenza specific IgG levels by 6 to 8 months of age, which was not, however, comparable in all infants. In contrast, most of the infants showed an increase in IgA responses throughout the first year of life

**Conclusions:**  This new analytical method can be applied in a larger study to understand the impact of maternal imprinting on influenza immunity.

## Introduction

The immune system in neonates and young infants is initially immature, without adequate protection against infections. The dogma is that during infancy, systemic protection is passively provided by transplacental transfer of IgG antibodies and oral and gastrointestinal mucosal protection via breast milk (BM) containing predominantly IgA and some IgG. However, the clinical protection provided by maternal influenza immunization or exposure varies by season and the corresponding match against circulating influenza strains. Therefore, maternal influenza exposure, whether through immunization or natural infection, provides maternal protection and has the potential to imprint the infant immune system and significantly impact infant morbidity and mortality, as recently comprehensively reviewed ^1^.

Maternal influenza immunization prior to or during pregnancy provides clinical protection with 70% efficiency in the infant ^2– 5^. Only one study has described IgA antibody levels in human milk to influenza ^6^. Their results suggested that vaccination using a single strain of influenza A (A/New Caledonia/20/1999, H1N1) induced significantly higher IgA antibody levels than those seen in non-vaccinated, and those antibodies were positively correlated with viral neutralization. In addition, higher rates of exclusive breastfeeding in the first 6 months of life were associated with protection against febrile respiratory illness in the infants of vaccinated mothers, suggesting mucosal protection against influenza by BM antibodies. However, there is little data on how influenza antibody levels, or strain-specific antibody profiles, vary between mother’s serum, BM and infant serum. Data on the kinetic changes in anti-influenza IgG profiles between mother-infant pairs are also largely lacking.

In this pilot study utilizing a novel multiplex assay, we assessed infant immunity to various influenza strains reflecting maternal anti-influenza IgG levels and profiles in serum, as well as characterized IgA antibody responses in breast milk, which are distinct and reflect mucosal immunity. This new analytical method was applied to a small number of samples showing feasibility and patterns suggestive that a larger study needs to be done to understand the impact of maternal imprinting on influenza immunity.

## Methods

We utilized stored frozen human foremilk collected in the morning between hours 8 and 11 and 1–2 hours after the last feeding, from a prospective birth cohort recruited in 1997–2001 in Finland to assess immune factors in human milk ^7^. As part of this study, breast milk and serum samples were collected during follow-up visits, and included colostrum and breast milk at 1 month, 3 months, 6 months, 9 months, and 12 months of duration of lactation. However, a few samples were never received due to missed visits or sample timing had to be moved due to illness or other difficulties in getting to the scheduled visits, and some samples have been used up in prior studies. In the present study, the earliest available BM and a paired, timed serum sample was assessed from each of 7 mothers; ranging from 3 days to 2 months post-partum. Paired infant serum samples were assayed at up to three time points during the first 12 months of life, one prior to assumed disappearance of transplacentally transferred IgG, and one after. The samples collected in this cohort have been stored at -80°C with no recurrent freeze-thaw cycles. Aliquots have successfully been used in the past for measurement of serum and BM antibody levels with good antibody levels detected both for IgG and IgA ^7^. These mothers were unvaccinated, as guidelines for maternal influenza immunization were not in place at the time samples were collected. None of the infants had been vaccinated to influenza. Clinical characteristics and timing of samples available are shown in Table 1. The study was approved by the institutional review boards of the Helsinki University Central Hospital, the City of Helsinki, and the University of Rochester Medical Center, Rochester, NY.

**Table 1.** Mother-infant pairs, demographics and time of sampling.

Dyad	Maternal age (years)	Breast milk sample	Infant age at serum sample (month of collection)	Exclusive BF length	Total BF length
1	32	3 weeks	2 month (Nov) 8 month (May) 12 month (Aug)	4 months	nk
2	31	3 days	1 month (Feb) 13 month (Feb)	2.75 months	8.25 months
3	32	2 months	4 month (July) 7 month (Oct)	0 months	>7 months
4	32	3 days	2 month (July) 4 month (Sep) 6 month (Nov)	0 months	6 months
5	26	1 month	5 month (Sep) 7 month (Nov)	0 months	>7 months
6	23	4 days	3.5 month (Aug)	3.5 months	nk
7	30	5 days	2 month (July) 4 month (Sep) 6 month (Nov)	0 months	2 months

nk, not known; BF, breastfeeding.

We have developed a multiplex assay (mPlex-Flu) that simultaneously measures absolute antibody concentrations against up to 50 influenza strains ^8^. The mPlex-Flu assay has several advantages over the traditional hemagglutinin inhibition (HAI) titer assay: a linear readout over 4 logs, and high sensitivity. Our previous studies also showed that mPlex-Flu assay results highly consistent with the results from HAI and ELISA assays in human pre-and post-influenza vaccine study ^8^. In the present study, a panel of 30 individual recombinant hemagglutinin (rHA) proteins of influenza virus strains and chimeric rHAs were used (see Table 2). This allowed us to estimate the specific anti-influenza IgG and IgA levels against H1, H2, H3 and Flu B seasonal influenza strains, as well as HA stalk specific antibodies using chimeric rHA (i.e. head from one influenza strain and stalk from another strain), cH5/1 and cH9/1 specific for group 1 (i.e. H1, H2, H5, H6) and cH4/7 and cH5/3 for group 2 (i.e. H3, H7) influenza strains, as previously described ^8^. The mPlex-Flu assay has been shown to strongly correlate with functional assays of influenza specific antibodies, including the hemagglutination inhibition (HAI) and influenza virus micro-neutralization assays ^8, 9^. In addition, we have previously shown that the mPlex-Flu assay has excellent strain specificity using competitive binding studies ^8^, which we also used here to confirm our results in human breast milk samples ( Supplementary Figure 1). These data suggest minimal non-specific binding in paired samples. As we and others have previously demonstrated, there is cross-reactivity of anti-HA antibodies between antigenically similar influenza strains based on similar or shared epitopes.

**Table 2.** The HA panel of the mPlex-Flu assay.

Influenza Virus Type	Subtypes	Full Name of Viruses	Abbreviation	Genbank Accession #	Bead Region
A	H1	A/South Carolina/01/1918	A/SC18	AF117241.1	35
		A/PR/8/34	A/PR8	CY148243.1	37
		A/USSR/1977	A/USSR77	DQ508897.1	55
		**A/Texas/36/91**	A/Tex91	DQ508889.1	43
		**A/New Caledonia/20/1999**	A/NewCal99	CY125100.1	18
		**A/California/07/2009**	A/Cali09	FJ966974.1	54
	H2	A/Japan/305/1957	A/Jap57	L20407.1	19
	H3	A/HongKong/1/1968	A/HK68	CY009348.1	53
		A/Port Chalmers/1/1973	A/PC73	CY112249.1	52
		**A/Perth/16/09**	A/Per09	GQ293081.1	47
		**A/Victoria/361/11**	A/Vic11	KM821347	56
		**A/Texas/50/2012**	A/Tex12	KC892248.1	36
		**A/Switzerland/2013**	A/Swi13	EPI537866	22
	H5	A/Viet Nam/1203/2004	A/Viet04	EF541403	30
	H6	A/chicken/Taiwan/67/2013	A/TW13	KJ162860.1	33
	H7	A/rhea/North Carolina/39482/1993	A/rheaNC93	KF695239	39
		A/Shanghai/1/2013	A/SH13	KF021597.1	44
	H9	A/guinea fowl/Hong Kong/WF10/1999	A/gfHK99	AY206676.1	42
B		**B/Malaysia/2506/2004**	B/Maly04	CY040449.1	51
		**B/Brisbane/60/2008**	B/Bris08	CY115343	46
		**B/Wiscosin/01/2010**	B/Wis10	KC306166.1	45
		**B/Massachustts/2/2012**	B/Mass12	KF752446.1	65
HA domains		Head of A/duck/Czech/1956	H4 Head		30
		Head of A/Shanghai/1/2013	H7 Head		25
		head of A/Indonesia/5/05	H5 Head		26
		Head of A/guinea fowl/Hong Kong/ WF10/1999	H9 head		48
Chimeric HA		cH5/1 (A/Indonesia/5/05, A/California/07/2009)	cH5/1Cal09		27
		cH9/1 (A/gf/HK/WF10/1999, A/California/07/2009)	cH9/1Cal09		20
		cH5/1 (A/Indonesia/5/05, A/Perth/16/09)	cH5/3		28
		cH4/1 (A/duck/Czech/1956, A/Shanghai/1/2013)	cH4/7		29

Seasonal Vaccine strains in Bold Different colors determine the influenza virus type or subtypes. Influenza B virus showed in Green. Bark blue, gray, and light blue, purple and dark green present H1, H2, H5, H6 and H9 subtype influenza viruses, respectively, HA phylogenic group 1 viruses. Red and orange present H3 and H7, HA phylogenic group 2 viruses. Other black present the HA1 domains of HA or chimeric HAs.

In the present study, samples of maternal serum (diluted 1:500 for IgA and 1:5000 for IgG), infant serum (1:10) and BM (1:10) were diluted using PBS and incubated with rHA coupled Luminex beads (Luminex Corp, Austin, TX). IgG or IgA binding was detected with anti-human IgG or IgA specific secondary antibodies (SouthernBiotech, AL, Cat No 2040-09, 2050-09, respectively). Median fluorescence intensities (MFI) were measured using a MAGPIX multiplex reader (Luminex Co.,TX) and converted into absolute IgG concentrations (ng/mL) using a IgG standard curve generated with a human standard serum, a mixture of sera from four subjects containing high levels of anti-influenza HA IgG and IgA against multiple influenza strains ^8^. Since serum IgA is monomeric, while BM secreted IgA (SIgA) is dimeric ^10^, the standard curves of BM SIgA against influenza viruses are very different from that of serum standard curves generated from our human standard serum sample. We thus report the magnitude of BM IgA anti-influenza HA antibody levels in MFI units. For consistency, and to allow direct comparison, we also report IgG levels in MFI units. All data were analysis by Prism 7, and heatmap figures were generated by Mathematic 11.2.

## Results

**Figure 1.**
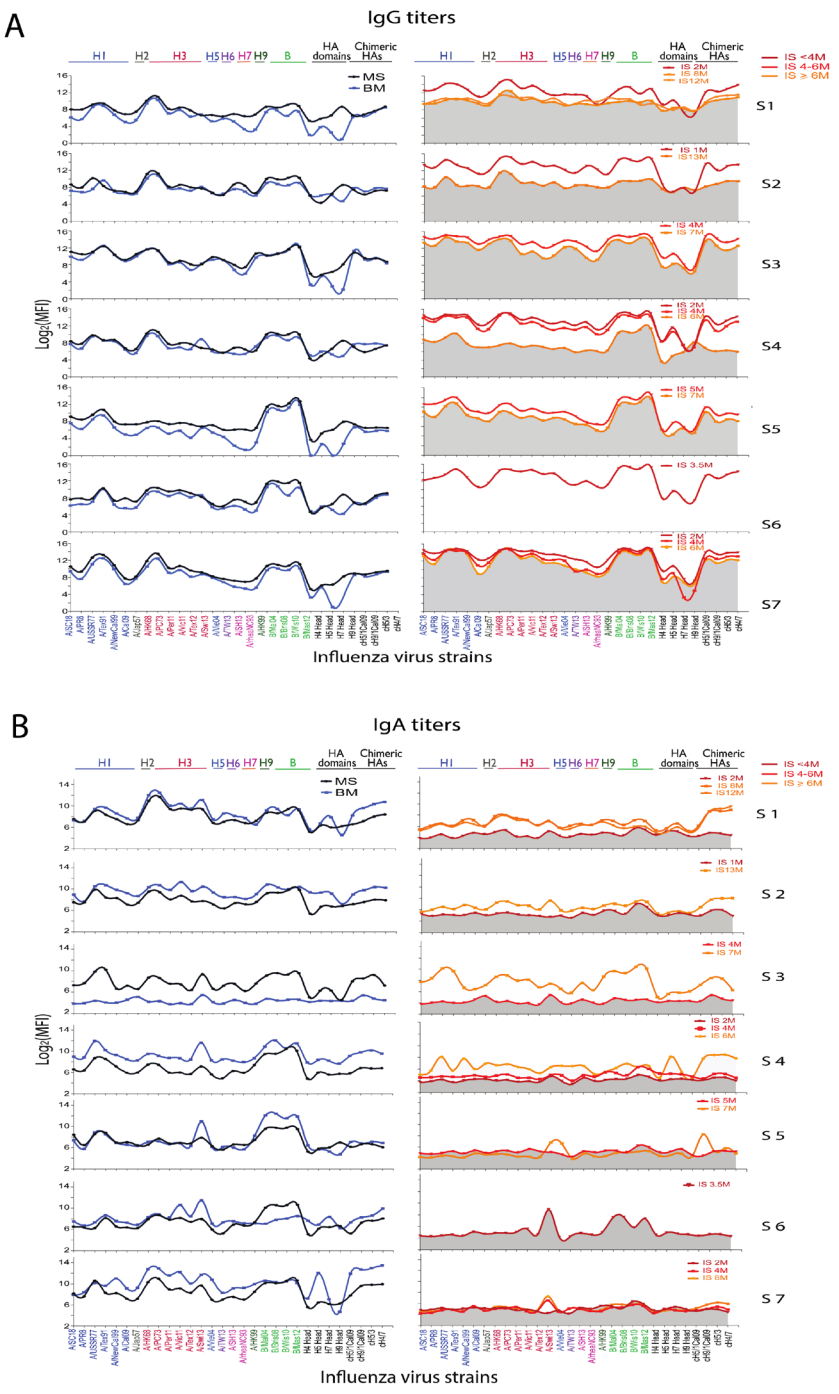
Influenza virus hemagglutinin (HA)-specific antibody IgG and IgA levels in paired mother-infant samples. The antibody levels against homologue and cross-reactive HA proteins from different influenza virus strains were evaluated by the mPlex-Flu assay using paired mother’s serum (MS), breast milk (BM) and infant’s serum (IS) at different ages expressed as months. **A.** The IgG antibodies against individual HA of influenza virus strains. Maternal serum was diluted 1:5000, infant serum 1:10 and breast milk 1:10. The IgG antibodies against influenza virus HA were estimated using Phycoerythrin (PE)-conjugated anti-human IgG (γ chain specific) secondary antibodies (SouthernBiotech, AL) and shown as means of median fluorescence intensity (MFI) (n=3). The antibody titers (Log _2_MFI) against individual rHA of influenza virus strains were plotted and connected by LOWESS curves. In the panel of IgG MFI units of infant serum samples, the gray is the area under HA antibody curve of oldest sampling time point in the same subject. **B.** The IgA antibodies against individual HA of influenza virus strains. Maternal serum was diluted 1:500, infant serum 1:10 and breast milk 1:10. Then IgA antibodies were detected using PE-conjugated anti-human IgA (α chain specific) secondary antibodies (SouthernBiotech, AL) and shown as the mean of median fluorescence intensity (MFI) (n=3). The antibody titers (Log _2_MFI) against individual rHA of influenza virus strains were plotted and connected by LOWESS curves. In the panel of Ig MFI units of infant serum samples, the gray is the area under the HA antibody curve of the youngest time point in the same subject.

**Figure 2.**
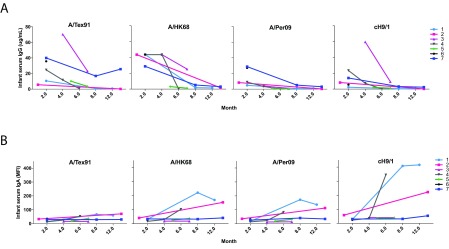
IgG antibody concentrations against selected influenza virus hemagglutinin (HA) in infants’ sera. **A.** IgG antibody levels against selected influenza virus HA were plotted over time during first year (infant age in months) for 7 infant subjects from Figure 1A data. The specific antibody concentration of IgG is shown as the means for triplicates (n=3). **B.** The IgA antibody levels against selected influenza virus HA in infant sera were plotted over time during first year for 7 infant subjects from Figure 1B data. The specific antibody levels of IgA are shown as the means for MFI units (n=3).

This new analytical method was applied to a small number of samples showing feasibility and several interesting patterns. BM had a pattern of IgG reactivity very similar to maternal serum. Also, the levels and strain reactivity patterns of anti-influenza IgG in mother’s serum matched that of her infant, suggesting a robust transplacental transfer of antibodies. As expected, there was a steady decay of infant influenza specific IgG levels by 6 to 8 months of age ( Figure 1A). This decay was, however, not comparable in all infants. Interestingly, mothers with highest anti-influenza HA IgG antibodies had infants with high initial anti-HA antibody maintained until 6 months of age (Pairs #3 and #7), compared to a mother with the lowest initial IgG (Pair #4), suggesting that initial levels attained transplacentally are directly associated with the rate of decline of passive systemic immunity. By the end of the first year, infant #1 maintained 6-month IgG antibody levels, which is likely due to new, natural exposure. Supplementary Figure 2 shows the heatmaps of IgG and IgA antibodies to influenza strains measured by multiplex array in paired mother’s serum (MS), breast milk (BM) and infant serum (IS) samples. Figure 2A shows the trajectory of IgG antibodies to selected individual strains.

Unlike with IgG antibodies, BM influenza virus HA-specific IgA antibody levels and patterns differed from serum IgA reactivity patterns, when visually comparing the pattern of antibody reactivity. Only three mother-infant pairs showed high a degree of concordance (Pairs #1, #4, and #5). This may be due to the mucosal homing of IgA producing antibody-secreting cells to the mammary gland, resulting in a different antibody profile in breast milk from serum. Very low serum IgA antibodies in infants are consistent with the fact that IgA does not cross the placenta ( Figure 1B). The pattern of IgA anti-HA antibody binding was largely similar to that of mother’s serum and milk IgG, and predominantly against H1, H3 and B influenza strains. As opposed to infant IgG responses, most of the infants (Pairs #1–4) showed an increase in IgA responses throughout the first year of life ( Figure 1B), whereas no matching IgG antibody response was seen. This may be due to natural, mucosal exposure to influenza inducing local responses, possibly in the absence of a systemic infection inducing IgG antibodies. Figure 2B shows the trajectory of IgA antibodies to a few individual strains. Both anti-stalk group 1 (cH5/1, cH9/1) and group 2 (cH5/3 and cH4/7) IgG ( Figure 1A) and IgA antibodies ( Figure 1B), which can confer cross-strain immunity, were abundant in the early months in infant serum and BM, respectively.

Raw data for the present study Click here for additional data file. Copyright: © 2019 Järvinen KM et al. 2019 Data associated with the article are available under the terms of the Creative Commons Zero "No rights reserved" data waiver (CC0 1.0 Public domain dedication).

## Discussion

Our pilot data suggest feasibility for measuring antibody responses to influenza strains in maternal breast milk and paired infant serum utilizing a novel multiplex assay to assess changes over time. We show an anticipated decline in IgG responses to influenza HA in the first 5 months of life reflecting waning passive transplacentally acquired immunity, whereas the systemic IgA response in infants appears to be relatively poor, consistent with no vertical transfer. This does not exclude the possibility that such young infants might have a response at mucosal surfaces, such as in saliva upon exposure. Throughout the first year, however, a small increase in IgA antibodies, but not IgG antibodies, is seen in these unvaccinated infants, possibly suggesting that the adaptive immune response to natural exposure, in the absence of systemic infection induces initially local IgA, but not IgG antibodies. We also show that while the IgG specificity patterns were rather similar between breast milk and maternal serum, the patterns for IgA specificity were distinct and more pronounced in BM than those seen in serum. These data suggest that during this time, breast milk IgA may indeed be an important means of providing mucosal protection, which closely reflects maternal mucosal exposure to a variety of influenza strains to benefit the infant. Our previous results comparing food-specific IgA in human milk and maternal serum have shown similarly marked differences between human milk and serum IgA antibody profiles ^7^. This is likely reflecting the fact that IgA-producing cells in mammary gland originate in the gut- and bronchus-associated lymphoid tissue, which constitute an important defense mechanism of the newborn ^11^.

Although our study does not address the antibody profiles in vaccinated dyads, maternal influenza vaccination is recommended during pregnancy to induce infant post-partum passive immunity, and for infants after 6 months of age, although many choose to defer vaccination. As indicated by our pilot study, responses in mothers and infants are heterogeneous. At present, there is no robust literature or clinical method to optimize the vertical transfer of protective antibodies. Furthermore, the mechanisms of imprinting or maternal imprinting of the infant immune system are incompletely understood. Thus, there is a critical need for empirical data regarding maternal (serum, BM) and infant (serum) influenza-specific antibody levels over time to inform about maternal impact of influenza immunity, and to predict the individual window of infant susceptibility to influenza. Larger studies are required to further elucidate the interesting findings of this pilot study to aid in assessment of (maternal) imprinting of influenza immunity. Knowing the scope of passive immunity, both transplacental and that provided by BM, and when it vanishes, would allow for precision maternal-fetal and infant vaccination schedule design, also accounting for circulating influenza strains, seasonality, and vaccination status.

## Data availability

The data referenced by this article are under copyright with the following copyright statement: Copyright: © 2019 Järvinen KM et al.

Data associated with the article are available under the terms of the Creative Commons Zero "No rights reserved" data waiver (CC0 1.0 Public domain dedication). 

**F1000Research: Dataset 1.** Raw data for the present study, https://doi.org/10.5256/f1000research.16717.d224137 ^12^, including the following files:

- **Influenza-specific IgA antibody data as MFI.** The file contains the IgA antibody data expressed as MFI for a panel influenza strains generated by mPlex-Flu assay utilizing all breast milk and serum samples. (IgA_20160908_MFI.xlsx)
- **Influenza-specific IgG antibody data as MFI.** The file contains the IgG antibody data expressed as MFI for a panel influenza strains generated by mPlex-Flu assay utilizing all breast milk and serum samples. (IgG_20160908_MFI.xlsx)
- **Comparison of influenza-specific IgA antibodies between paired samples.** The MFI titer comparison of IgA antibody of maternal serum (MS) vs breast milk (BM) and infant’s serum (IS) over time using the Prism 7 software. (IgA version2018.pzfx)
- **Comparison of influenza-specific IgG antibodies between paired samples.** The file contains MFI unit comparison of influenza-specific IgG antibodies of maternal serum (MS) vs breast milk (BM) and infant’s serum (IS) over time using the Prism 7 software. (IgG version2018.pzfx)
- Program code for IgA heatmap. The Mathematica 2 program code for generation of the heatmap figure of IgA data of maternal serum (MS), breast milk (BM) and infant’s serum (IS) from mPlex-Flu assay. (IgA MFI Revised.nb)
- **Program code for IgG heatmap.** The Mathematica 2 program code for generation of the heatmap figure of IgG data of maternal serum (MS), breast milk (BM) and infant’s serum (IS) from mPlex-Flu assay. (IgG MFI Revised.nb)

## Supplementary material

**Supplementary Figure 1. The specificity of mPlex-Flu assay for determination of IgG/IgA antibodies against influenza viruses in human breast milk samples.** The specificity of mPlex-Flu assay determined by rHA protein pre-adsorption. The milk IgG or IgA antibody binding with/without rHA pre-absorption in mPlex-Flu assay is shown. The mean binding (MFI) showed in heatmap (n=4). We determine the specificity of mPlex-assay by using the antibody pre-adsorption assay as we previously descripted ^8^. Briefly, breast milk samples (1:10 dilution, within the detectable range of mPlex assay) were pre-incubated with individual recombinant HA (rHA) protein of influenza virus 0.25 μg for 1 hour for pre-absorption of the HA specific antibodies in breast milk; then run the mPlex-Flu assay to show the antibody binding with/without pre-absorption of A/Texas/36/1991 (A/Tex91, H1), A/Victoria/361/2011 (A/Vic11, H3), B/ B/Massachusetts/2/2012 (B/Mass12, B influenza), respectively. The results showed Pre-absorption with rHAs of A/Vic11 significantly inhibited two H3 strains in IgA, and all 5 H3 strains in IgG testing of breast milk samples. Those H3 strains are antigenic similar to A/Vic11, but did not affect IgG and IgA antibody binding with other influenza virus strains. Similar results were observed when milk samples were pre-absorbed by rHA of B/Mass12 and A/Tex91. Those results suggested a high type or subtype specificity of the mPlex-Flu assay for determination of human breast milk IgA and IgG anti-influenza strain specificity.

**Supplementary Figure 2. Heatmaps of IgG and IgA antibodies to influenza strains measure by multiplex array in paired mother’s serum (MS), breast milk, (BM) and infant serum (IS) samples.** The antibody levels against homologue and cross-reactive HA proteins from different influenza virus strains were evaluated by the mPlex-Flu assay using paired mother’s serum, breast milk (BM) and infant’s serum at different ages expressed as months. A. The IgG antibodies against individual HA of influenza virus strains. MS samples were diluted 1:5000, BM samples were diluted 1:10, and IS samples were diluted 1:10. The IgG antibodies against influenza virus HA were estimated using Phycoerythrin (PE)-conjugated anti-human IgG (γ chain specific) secondary antibodies (SouthernBiotech, AL) and shown as mean median fluorescence intensity (MFI) (n=3). B. The IgA antibodies against individual HA of influenza virus strains. MS samples were diluted 1:500, BM samples were diluted 1:10 and IS samples were 1:10 diluted. Then IgA antibodies were detected using PE-conjugated anti-human IgA (α chain specific) secondary antibodies (SouthernBiotech, AL) and shown as MFI unit also (n=3).

## References

[1] Marchant A Sadarangani M Garand M: Maternal immunisation: collaborating with mother nature. *Lancet Infect Dis.* 2017;17(7):e197–e208. 10.1016/S1473-3099(17)30229-3 28433705

[2] Manske JM: Efficacy and effectiveness of maternal influenza vaccination during pregnancy: a review of the evidence. *Matern Child Health J.* 2014;18(7):1599–609. 10.1007/s10995-013-1399-2 24272875

[3] Zaman K Roy E Arifeen SE: Effectiveness of maternal influenza immunization in mothers and infants. *N Engl J Med.* 2008;359(15):1555–64. 10.1056/NEJMoa0708630 18799552

[4] Madhi SA Cutland CL Kuwanda L: Influenza vaccination of pregnant women and protection of their infants. *N Engl J Med.* 2014;371(10):918–31. 10.1056/NEJMoa1401480 25184864

[5] Tapia MD Sow SO Tamboura B: Maternal immunisation with trivalent inactivated influenza vaccine for prevention of influenza in infants in Mali: a prospective, active-controlled, observer-blind, randomised phase 4 trial. *Lancet Infect Dis.* 2016;16(9):1026–35. 10.1016/S1473-3099(16)30054-8 27261067 PMC4985566

[6] Schlaudecker EP Steinhoff MC Omer SB: IgA and neutralizing antibodies to influenza a virus in human milk: a randomized trial of antenatal influenza immunization. *PLoS One.* 2013;8(8):e70867. 10.1371/journal.pone.0070867 23967126 PMC3743877

[7] Seppo AE Savilahti EM Berin MC: Breast milk IgA to foods has different epitope specificity than serum IgA-Evidence for entero-mammary link for food-specific IgA? *Clin Exp Allergy.* 2017;47(10):1275–84. 10.1111/cea.12945 28449395 PMC6693637

[8] Wang J Hilchey SP Hyrien O: Multi-Dimensional Measurement of Antibody-Mediated Heterosubtypic Immunity to Influenza. *PLoS One.* 2015;10(6):e0129858. 10.1371/journal.pone.0129858 26103163 PMC4478018

[9] Wang J Hilchey SP DeDiego M: Broad cross-reactive IgG responses elicited by adjuvanted vaccination with recombinant influenza hemagglutinin (rHA) in ferrets and mice. *PLoS One.* 2018;13(4):e0193680. 10.1371/journal.pone.0193680 29641537 PMC5894995

[10] Fagarasan S Kinoshita K Muramatsu M: *In situ* class switching and differentiation to IgA-producing cells in the gut lamina propria. *Nature.* 2001;413(6856):639–43. 10.1038/35098100 11675788

[11] Peri BA Theodore CM Losonsky GA: Antibody content of rabbit milk and serum following inhalation or ingestion of respiratory syncytial virus and bovine serum albumin. *Clin Exp Immunol.* 1982;48(1):91–101. 7200842 PMC1536582

[12] Järvinen KM Wang J Seppo AE: Dataset 1 in: Novel multiplex assay for profiling influenza antibodies in breast milk and serum of mother-infant pairs. *F1000Research.* 2018 10.5256/f1000research.16717.d224137 PMC6419979 30918628

