# Supplementary figures and images for: Novel multiplex assay for profiling influenza antibodies in breast milk and serum of mother-infant pairs
Source: F1000Res. 2019 Mar 11;7:1822. Originally published 2018 Nov 20. [Version 2] doi: 10.12688/f1000research.16717.2 (PMC6419979; doi:10.12688/f1000research.16717.2)

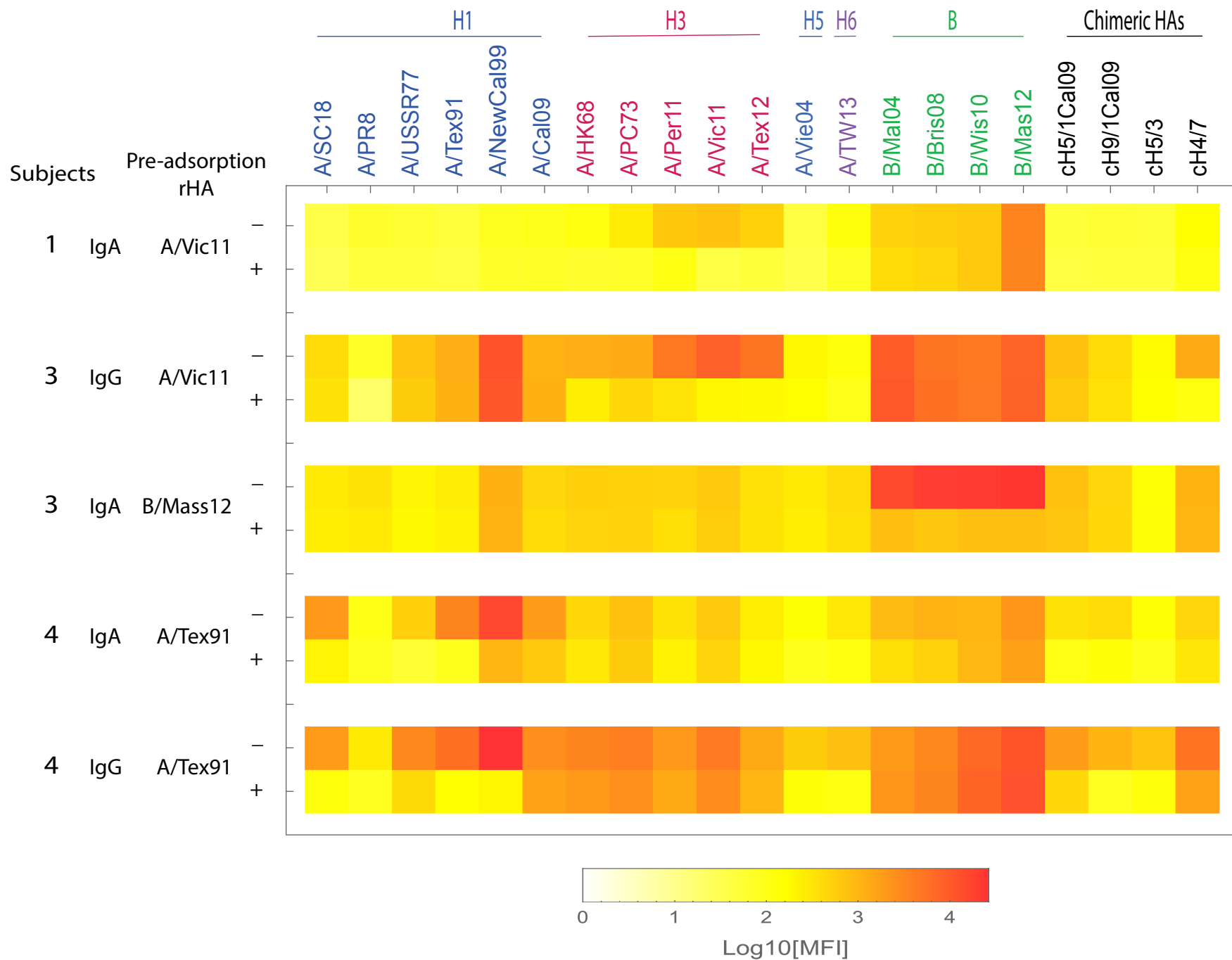

Supplement: Supplementary file 2 [file f1000research-7-20083-s0001.tgz › e985eb36-c2b1-4bb4-9439-4d0f62282873_Supplementary_figure_1_v2.pdf]

A

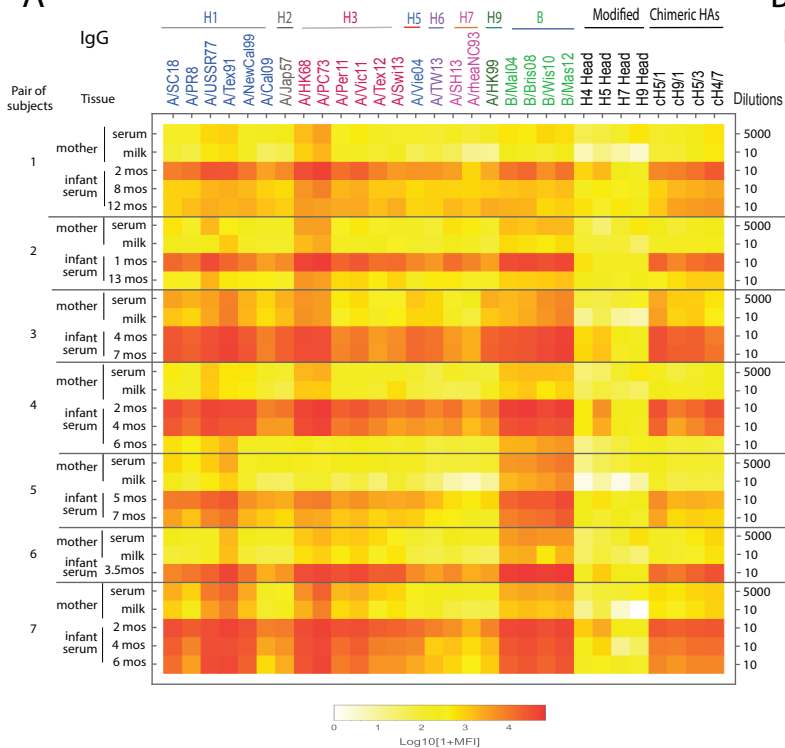

B

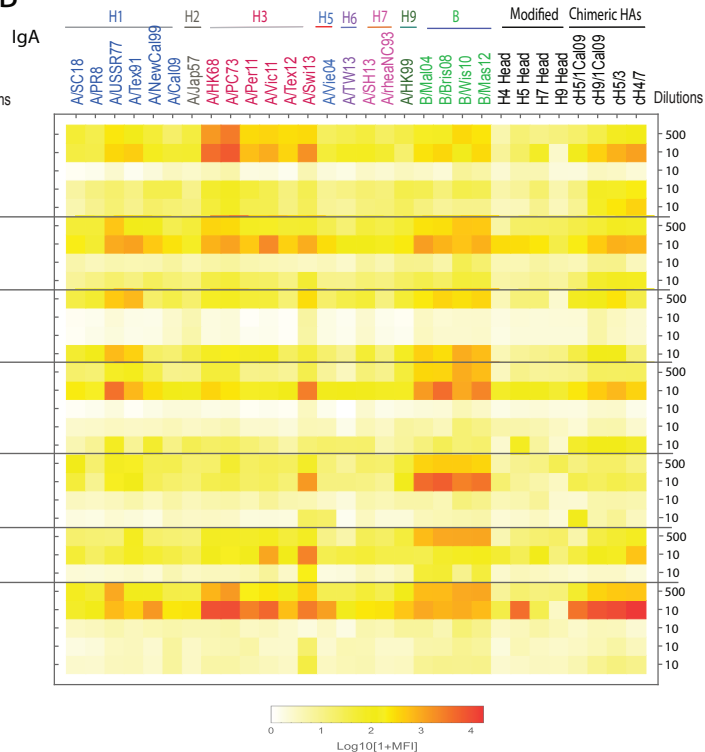

Supplement: Supplementary file 3 [file f1000research-7-20083-s0002.tgz › cb2f5bd0-7dcb-42cc-89e7-d886cb308fd8_Supplementary_figure_2_v2.pdf]
