# Supplementary material for: Novel multiplex assay for profiling influenza antibodies in breast milk and serum of mother-infant pairs
Source: F1000Res. 2019 Mar 11;7:1822. Originally published 2018 Nov 20. [Version 2] doi: 10.12688/f1000research.16717.2 (PMC6419979; doi:10.12688/f1000research.16717.2)
Supplement: Peer review history

# Peer review: Reviewer response for version 2

**DOI:** 10.5256/f1000research.20083.r45556

**Published:** 2019-03-13

**Authors:** Kirsty Le Doare

**Affiliations:** 1 St George's University of London, London, UK

The queries have been answered.

I have read this submission. I believe that I have an appropriate level of expertise to confirm that it is of an acceptable scientific standard.

# Peer review: Reviewer response for version 1

**DOI:** 10.5256/f1000research.18274.r43531

**Published:** 2019-02-04

**Authors:** David C. Dallas, Jiraporn Lueangsakulthai

**Affiliations:** 1 Nutrition Program, School of Biological and Population Health Sciences, College of Public Health and Human Sciences, Oregon State University, Corvallis, OR, USA; 2 Nutrition Program, School of Biological and Population Health Sciences, College of Public Health and Human Sciences, Oregon State University, Corvallis, OR, USA

Overall, this is a very interesting method and an interesting application. I have a few comments.

 Page 1, Methods: This should be “which can provide mucosal protection”.

 Page 3, Methods: Shouldn’t it be -80 Celsius? Not Fahrenheit.

 Page 3, Methods: Please specify how the dilutions used for each sample type were determined.

 Page 4, Table 2: Need to specify the meaning of the coloring of the text. It is not clear why some are colored and some are not. The table only says that “seasonal vaccine strains are in bold” but does not mention the colors.

 Page 5, Results, paragraph 2: How do you defined “concordance”? What are your thresholds for how similar it needs to be? Pair 4 (IgA titers) could also be argued to be concordant.

 Page 6,  Figure 1 legend: curvel -> curve? Curvel is written twice—not sure if this is a word I’m not familiar with or a misspelling of “curve”.

 Page 6, Figure 1: The text is very small. Can you figure out how to enlarge please?

 Page 6, Figure 1 legend: MS abbreviation needs to be defined in the legend.

 Page 6, Figure 1 legend: “IS”, infant serum needs to be defined in legend.

 Page 6, Figure 1 legend: Need to explain why Log2 was selected for this display.

 Page 6, Figure 1 legend: Need to explain what “net” MFI refers to. What is the “net” part?

 Page 6, Figure 1 legend: Unclear why Log2(MFI + 1) is used. What is the + 1 for?

We have read this submission. We believe that we have an appropriate level of expertise to confirm that it is of an acceptable scientific standard.

# Response

**Published:** 2019-03-11

**Authors:** Kirsi Jarvinen

**Affiliations:** University of Rochester Medical Center, USA

Overall, this is a very interesting method and an interesting application. I have a few comments.

COMMENT: Page 1, Methods: This should be “which can provide mucosal protection”.

REPLY: corrected.

COMMENT: Page 3, Methods: Shouldn’t it be -80 Celsius? Not Fahrenheit.

REPLY: corrected.

COMMENT: Page 3, Methods: Please specify how the dilutions used for each sample type were determined.

REPLY: Dilutions were determined by pilot testing to ensure IgG and IgA influenza virus-specific antibody binding within the mPlex-Flu assay range, i.e. the detectable range of the mPlex-Flu assay (the lower to the upper limits of quantification (LLOQ and ULOQ) concentrations for each influenza HA and each Ig isotype) (Wang et al 2018, ref #8). The pilot testing had been performed to confirm the dilution of samples enables all samples in this detectable range. Usually, the dilutions of samples are not laborious to be determined since mPlex-Flu can detect four Log10 scales.

COMMENT: Page 4, Table 2: Need to specify the meaning of the coloring of the text. It is not clear why some are colored and some are not. The table only says that “seasonal vaccine strains are in bold” but does not mention the colors.

REPLY: The color determines the subtype of influenza viruses. We added the notes under the table to make it clear.

COMMENT: Page 5, Results, paragraph 2: How do you defined “concordance”? What are your thresholds for how similar it needs to be? Pair 4 (IgA titers) could also be argued to be concordant.

REPLY: By visually comparing the patterns of antibody reactivity. We agree that #4 is also concordant and have revised the manuscript accordingly.

COMMENT: Page 6,  Figure 1 legend: curvel -> curve? Curvel is written twice—not sure if this is a word I’m not familiar with or a misspelling of “curve”.

REPLY: should say curve. Corrected.

COMMENT: Page 6, Figure 1: The text is very small. Can you figure out how to enlarge please?

REPLY: We have increased the font on those figures. Thank you for pointing out.

COMMENT: Page 6, Figure 1 legend: MS abbreviation needs to be defined in the legend.

REPLY: Done

COMMENT: Page 6, Figure 1 legend: “IS”, infant serum needs to be defined in legend.

REPLY: Done

COMMENT: Page 6, Figure 1 legend: Need to explain why Log2 was selected for this display.

REPLY: Log2 is customarily used for antibody titers to indicate fold-change.

COMMENT: Page 6, Figure 1 legend: Need to explain what “net” MFI refers to. What is the “net” part?

REPLY: It should say MFI, not net MFI. We have removed “net”.

COMMENT: Page 6, Figure 1 legend: Unclear why Log2(MFI + 1) is used. What is the + 1 for?

REPLY: It should be Log _2_MFI. Corrected in the manuscript.

# Peer review: Reviewer response for version 1

**DOI:** 10.5256/f1000research.18274.r41719

**Published:** 2018-12-11

**Authors:** Kirsty Le Doare

**Affiliations:** 1 St George's University of London, London, UK

This interesting pilot study investigates the association between anti-influenza antibodies measured by Luminex in blood and breast milk.

 Breast milk is a much understudied body fluid. However, the numbers in this study and the fact that the timing of breast milk collection varies so greatly means that the results are merely descriptive. Breast milk changes throughout feeding, by time of day and by time of collection relative to feeds. Thus the sampling, although I understand the rationale of convenience, means that the results are not interpretable as they currently are described.

 It would be useful to add more detail from the original study on timing of sample collection etc.

 How do you account for different rates of decline? IgG rate of decline is uniform across many pathogens and I am unsure why this should be any different in your study. What was the half life and how does it compare to the other studies you cite?

 Could the differences you see between pairs also be due to non-specific binding within your assay?

I have read this submission. I believe that I have an appropriate level of expertise to confirm that it is of an acceptable scientific standard, however I have significant reservations, as outlined above.

# Response

**Published:** 2019-01-28

**Authors:** Kirsi Jarvinen

**Affiliations:** University of Rochester Medical Center, USA

COMMENT: Breast milk is a much understudied body fluid. However, the numbers in this study and the fact that the timing of breast milk collection varies so greatly means that the results are merely descriptive. Breast milk changes throughout feeding, by time of day and by time of collection relative to feeds. Thus the sampling, although I understand the rationale of convenience, means that the results are not interpretable as they currently are described.

 REPLY: As mentioned under methods, we utilized stored frozen foremilk collected in the morning. The sample was not a convenience sample but taken according to the study protocol using strict criteria in a prospective manner.  Specifically samples were foremilk (not mixed sampling or hindmilk) taken always between hours 8 and 11, and 1-2 hours after the last feeding.

 COMMENT: It would be useful to add more detail from the original study on timing of sample collection etc.

 How do you account for different rates of decline? IgG rate of decline is uniform across many pathogens and I am unsure why this should be any different in your study. What was the half life and how does it compare to the other studies you cite?

 REPLY: As part of the prospective study, breast milk and serum samples were collected during followup visits, and included colostrum and breast milk at 1 month, 3 months, 6 months, 9 months, and 12 months of duration of lactation. However, a few samples were never received due to missed visits or sample timing had to be moved due to illness or other difficulties in getting to the scheduled visits, and some samples have been used up in prior studies. Therefore, not all sample time points are available for all subjects. Regarding decline in IgG, we agree that the levels decline throughout the lactation, as seen in Fig 2a. Due to the small number of samples, no statistical modeling or halflife calculations have been done.

 COMMENT: Could the differences you see between pairs also be due to non-specific binding within your assay?

 REPLY: The mPlex-Flu assay is a novel assay to estimate the specific antibody binding to recombinant hemagglutinin (rHA) of multiple influenza virus strains. It has been shown to strongly correlate with all functional assays of influenza specific antibodies, such as hemagglutination inhibition (HAI) assay and influenza virus micro-neutralization assay (Wang J et al 2015 Ref 8, Wang J et al 2018 (new reference added #9)). In addition, in our previous study, mPlex-Flu assay had been shown to have excellent specificity for each influenza virus strains using the HA specific bind blocking in multiple plex assay (Ref 8), and we also used this method to confirmed the specificity of mPlex-Flu assay using human milk samples is similar to using human serum samples (New Supplementary Fig 1). These data suggest minimal non-specific binding between pair samples. As we have demonstrated before, there is cross-reactivity of anti-HA antibodies between antigenically similar influenza strains, which is directed against specific B cell epitopes with similar or homologous sequences and antigenic similarity.  This type of cross reactivity, while it may be broad, is specific and based on shared epitopes. 
